# Sodium Selenate Ameliorates Cardiac Injury Developed from High-Fat Diet in Mice through Regulation of Autophagy Activity

**DOI:** 10.1038/s41598-019-54985-3

**Published:** 2019-12-10

**Authors:** Shuqiang Zhang, Jialiang Xu, Zhisong He, Feng Xue, Tingbo Jiang, Mingzhu Xu

**Affiliations:** 1 Key Laboratory of Neuroregeneration of Jiangsu and Ministry of Education of China, Co-innovation Center of Neuroregeneration, Nantong University, Nantong, Jiangsu 226001 China; 2 Department of Cardiology, the First Affiliated Hospital of Soochow University, Suzhou, Jiangsu 215006 China

**Keywords:** Cardiac hypertrophy, Obesity

## Abstract

Obesity is often accompanied by dyslipidemia, high blood glucose, hypertension, atherosclerosis, and myocardial dysfunction. Selenate is a vital antioxidant in the cardiovascular system. The beneficial effects of selenate on obesity-associated cardiac dysfunction and potential molecular mechanism were identified in both H9C2 cells and C57BL/6J mice hearts. The cardiac histological preformation in C57BL/6J mice were evaluated by cross-sectional area (CSA) of cardiomyocytes and percent area of fibrosis in the left ventricles. The cardiac autophagy flux in H9C2 cells and C57BL/6J mice hearts was analyzed by Western blots and the number of autophagosomes and autolysosome in H9C2 cells. In the present study, we found that lipid overload caused increases in serum lipid, CSA, and percent area of fibrosis. We further found that lipid-induced accumulation of autophagosomes  was due to depressed autophagy degradation, which was not restored in the pretreatment with 3-methyladenine and chloroquine, whereas, it was improved by rapamycin. Moreover, we demonstrated that increased levels of serum lipid, CSA, percent area of fibrosis and mRNA expression related to cardiomyocytes hypertrophy and fibrosis were significantly reduced after selenate treatments of mice. We also found selenate treatment significantly down-regulated activity of the Akt pathway, which was activated in response to lipid-overload. Furthermore, selenate dramatically improved cardiac autophagic degradation which was suppressed after exposure to lipid-overload in both H9C2 cells and C57BL/6J mice hearts. Taken together, selenate offers therapeutic intervention in lipid-related metabolic disorders, and protection against cardiac remodeling, likely through regulation of the activity of autophagic degradation and Akt pathway.

## Introduction

Obesity is a high-risk factor that causes hypertension, diabetes, atherosclerosis, and other chronic diseases, all of which increase the rates of morbidity, mortality, and financial burden. Patients with obesity are likely to suffer from cardiovascular complications, such as left ventricular hypertrophy, cardiac fibrosis deposition, and atrial fibrillation^1–3^. The underlying molecular defect involved in the pathogenesis of metabolic cardiomyopathy and autophagic changes is not fully understood. Altered cardiac autophagy is a pivotal cause of obesity-induced disruption in cardiac structure and function^1,4,5^. Autophagy is a highly-conserved degradation pathway in which intracellular proteins and damaged organelles are delivered to and degraded in the lysosomes. Autophagy is also a tightly regulated and highly inducible process^6^. An inappropriately activated or suppressed autophagy pathway may result in cell injury or death. However, even if autophagy occurs in response to the same stress or under the same conditions, it is uncertain as to whether it will change in the same direction^7^, suggesting autophagy has distinct regulatory mechanisms. Autophagic marker Beclin-1 is an early promoter of autophagy and the conversion of LC3-I to LC3-II indicates the formation of autophagosomes^8^. The p62 protein is degraded destined for the lysosome, which is inversely associated with autophagy activity^9,10^. It has been demonstrated that the insulin/insulin-like growth factor receptor activates several signaling pathways such as phosphoinositide 3-kinase (PI3K)/protein kinase (Akt)/mammalian target of rapamycin (mTOR) and Akt/glycogen synthase kinase-3β (GSK3β)^11–14^. Akt is involved in the regulation of cell proliferation, survival, and metabolism; meanwhile, it also mediates in cardiac hypertrophy, interstitial fibrosis, as well as cardiac autophagy^11–17^.

Selenium is not an antioxidant on its own, but it is incorporated as an integral component of several antioxidant enzymes which were involved in maintaining cell survival, modulating cellular differentiation, protecting against oxidative damage and metabolic disorders^18^. Selenium is also a vital element in the cardiovascular system, as selenium deficiency has been linked to Keshan cardiomyopathy, the process of cardiac remodeling, and chronic heart failure, due to increased demand for antioxidant activity and/or insufficiency selenium intake^19,20^. Accumulating evidences confirmed that the recommendations for selenium might be required to increase anti-oxidative and anti-apoptotic effects, and contribute to protecting cardiomyocytes from hyperglycemia-induced heart damage, reducing cardiac remodeling, and improving cardiac dysfunction^18,19,21^; but also bring a benefit in the prevention of atherosclerosis in subjects with low selenium status^22^.

Sodium selenate, an oxidized form of selenium, has the profound effects on Alzheimer’s disease^23^, however, no study has investigated whether sodium selenate may be an effective dietary intervention for cardiac abnormalities due to hyperlipidemia exposure. Here, we evaluated therapeutic effects of sodium selenate (12 μg/mL sodium selenate added to the drinking water) on cardiac pathologic changes and autophagy disorders due to obesity using obese mouse models and cultured cells.

## Materials and Methods

### Cell culture, reagents, and treatments

H9C2 cells, a myoblastic cell line derived from embryonic BD1X rat myocardium, were obtained from the Cell Bank of Type Culture Collection of Chinese Academy of Sciences (Shanghai, China) and cultured in Dulbecco’s modified Eagle’s medium (DMEM, Gibco) supplemented with 10% fetal bovine serum (FBS, Gibco) in a humidified atmosphere of 5% CO_2_ at 37 °C. Various concentration of palmitic acid (PA, P5585, Sigma) in 1% fat-free bovine serum albumin (BSA, Roche) was prepared. The cells were treated with 0.4 mM PA for various time points or with various concentrations of PA for 12 hrs. For vehicle control, H9C2 cells were incubated with 1% fat-free BSA solution. Chloroquine (CQ, C6628, Sigma), 3-methyladenine (3-MA, HY-19312, MCE), sodium selenate (S8295, Sigma) were dissolved in phosphate buffer saline (PBS); and rapamycin (Rapa, HY-10219, MCE) and LY294002 (NSC 697286, MCE) was dissolved in DMSO. All reagents were diluted to the indicated concentrations.

### Cell viability assay

Cell viability was determined by using Cell Counting Kit-8 (CCK-8, Dojindo Molecular Technologies, Inc., Japan). H9C2 cells were seeded in 96-well culture plates and treated for a considerate time. Then, 10 μL CCK-8 were added to each well and incubated for 70 mins. Cell viability was estimated at 450 nm by manufacturer’s protocol.

### Cell cycle analysis

H9C2 cells were centrifuged, suspended in 70% ethanol, and fixed overnight at 4 °C. The cell cycle was detected using the Cell Cycle and Apoptosis Analysis Kit (Beyotime, P0010) according to the manufacturer’s protocol. The distribution of cells in G_0_/G_1_, S, and G_2_ phases was calculated using the ModFit LT^TM^ 32 software.

### Cell apoptosis assay

Cell apoptosis was detected by DeadEnd™ Fluorometric TUNEL System (G3250, Promega) according to the manufacturer’s instructions. H9C2 cells were cultured on slides and treated according to the experimental methodology. Then, samples were fixed, permeabilized, equilibrated, and added the mixture (containing Nucleotide Mix and rTdT Enzyme) for incubation at 37 °C. After the reactions were terminated, the sliders were detected with localized green fluorescence of apoptotic cells in a blue nuclear background by fluorescence microscopy.

### H9C2 cells expressing stubborn red fluorescent protein and sensitive green fluorescent protein fused to microtubuleassociated protein light chain 3 (stubRFP-sensGFP-LC3) and expressing GFP fused to p62 protein (GFP-p62)

In order to monitor autophagy flux of cells, we established a stable expression stubRFP-sensGFP-LC3 H9C2 cell line infected by directly adding lentivirus expressing stubRFP-sensGFP-LC3 fusion protein (GeneChem, LV-MAP1LC3B, 3905-1) at an MOI of 20 for 72 hours at about 70% infection efficiency. Since green fluorescence is quenched by acidic condition of lysosome, the puncta of green and red displays autophagosome puncta (yellow puncta, G + R+) and red puncta represents autophagolysosome puncta (red puncta, G − R+). The number of yellow puncta (autophagosome, G + R+) and red only puncta (autophagolysosome, G − R+) were detected by confocal microscopy. Similarly, we established a stable expressing GFP-p62 protein H9C2 cell line by infecting it with an adenovirus expressing GFP-p62 protein (Ad-GFP-p62, Beyotime, C3015) at an MOI of 30 for 24 hours with 70% infection efficiency analyzed by fluorescent microscopy. The area and number of green puncta were increased in cases of inhibited autophagy activity. After treatments, stable expressing stubRFP-sensGFP-LC3 or Ad-GFP-p62 H9C2 cells were washed with PBS, fixed with 4% paraformaldehyde, permeabilized, and stained with Hoechst 33342 (Invitrogen^TM^, USA). Images were acquired under a fluorescence microscope.

### Animal model

All laboratory animals were handled and maintained in accordance with the Guide for the Care and Use of Laboratory Animals as published by the US National Academies Press (Eighth Edition, update, 2011). The approved protocol for animal use was approved by the Committee on the Ethics of Animal Experiments of Nantong University.

**Figure 1:**
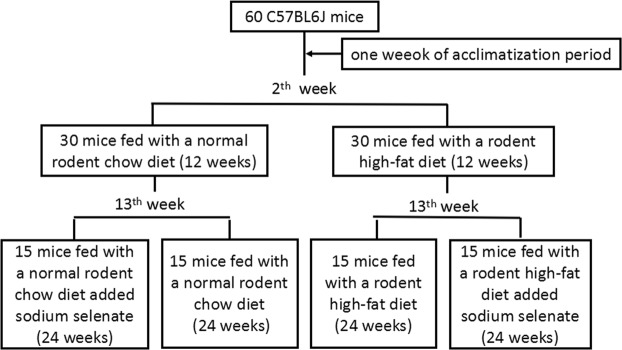
The whole course of feeding scheme.

Female C57BL/6J mice were purchased from Shanghai SLAC Laboratory Animal Center (Shanghai, China). Animals were housed under a constant room temperature at 25 ± 2 °C and 50 ± 5% humidity with a 12-hour daylight period and 12-hour darkness period, with free access to water. After an acclimatization period of one week, C57BL/6J mice were randomly divided into two weight-matched groups. Thirty mice were fed with the normal rodent chow diet, while the remaining 30 mice were fed with a rodent high-fat diet (HFD) with 60% kcal fat (Research Diets, Inc.™, catalog number: D12492, USA) for 12 weeks. In the thirteenth week, mice fed with the normal rodent chow diet were randomly divided into two groups: 15 mice fed with a normal rodent chow diet (ND group, n = 15) and 15 mice fed with a normal rodent chow diet added sodium selenate (ND + Se group, n = 15). These groups remained in place for 24 weeks. Also in the thirteenth week, mice fed with a HFD were randomly divided into two groups: 15 mice provided with a HFD continually (HFD group, n = 15), 15 mice provided with a HFD added sodium selenate for the next 24 weeks (HFD + Se group, n = 15) (Fig. 1). 12 μg/mL sodium selenate were added to the drinking water, which were referred to the great effects of sodium selenate published before^23^.

### Histological studies

Mouse hearts were removed after sacrifice, then weighed and placed in 4% formaldehyde for histological study. The tissues were fixed in paraffin, sectioned into 5 μm thickness at the level of the papillary muscles for each heart, and stained with Hematoxylin and eosin (H&E) and Masson’s trichrome (MT). The cardiomyocyte cross-sectional area (CSA) was measured in 30 cells from 10 random fields in each section stained with H&E. Color images were made at 400× total magnification using Leica DM2000 with Digital Camera (JD Smart V V3.3). Fibrotic changes were assessed by MT staining, which is defined as the percent area of myocardial interstitial fibrosis as calculated by the blue fibrosis area divided by the sum of the total myocardial area in 10 randomly-selected fields in each section. Color images were made at 200× total magnification. The analysis of collagen was quantitatively evaluated by image analysis software AxioVision (Zeiss, Oberkochen, Germany).

### Protein extraction and Western blot analysis

After treatments, the cells were washed, and lysed in RIPA lysis buffer (P0013, Beyotime, China) containing phosphatase and protease inhibitors. Heart tissue was homogenized in a lysis buffer (P0013B, Beyotime, China) containing phosphatase and protease inhibitors. The protein samples were resolved and separated by SDS-PAGE, and then transferred onto poly-vinylidene difluoride (PVDF) membranes (Millipore). The membranes were incubated in a blocking buffer, followed by incubation with primary antibodies against specific proteins overnight: Akt (#9272, Cell Signaling Technology [CST], USA), phospho-Akt (serine^473^, #9271, CST, USA), p62/SQSTM1 (#5114, CST, USA), LC3 (#4108, CST, USA), and GAPDH (sc-25778, Santa Cruz Biotechnology, USA). The primary antibodies were diluted 1:1000 in 5% fat-free milk in TBS buffer overnight. The membranes were washed and incubated with Peroxidase-AffiniPure Goat Anti-Rabbit IgG (111-035-114, Jackson ImmunoResearch). The target-expressed protein-antibody complex was visualized by the Pierce^**TM**^ ECL Western blotting Substrate (#32106, Thermo Scientific). Images with adequate exposure were acquired for densitometry by using the Multi-Gauge software V3.0 from Fujifilm.

### RNA extraction and quantitative real-time PCR

Total RNA was extracted from heart tissue using E.Z.N.A.® Total RAN Kit (R6834, Omega Bio-Tek, Inc.), and reverse transcribed to cDNA by SuperScript 1st Strand cDNA Synthesis kit (BU-304-01, Biouniquer, China). Quantitative real-time PCR was performed using SYBR Green I by Roche LightCycle® 96 Instrument. The expression level was normalized to the levels of GAPDH transcripts. The primer sequences used for quantitative real-time PCR: ANP, 5′-3′ GCGGTGTCCAACACAGAT, 5′-3′ CTTCCTCAGTCTGCTCACTC; BNP, 5′-3′ TGGATCTCCTGAAGGTGCTG, 5′-3′ TGCATCTTGAATTGCTCTGGA; β-MHC, 5′-3′ AGGCAACTGGAGGAGGAGGT, 5′-3′CAGCCTTGGCCTCTGTCT; Collagen-I, 5′-3′ C-CTCCTGGCAAGAATGGAGA, 5′-3′AGCTGTTCCAGGCAATCCAC; Collagen-III, 5′-3′ TGCTCCTGTGCTTCCTGATG, 5′-3′ GACCTGGTTGTCCTGGAAGG; GAPDH, 5′-3′ AGGTCGGTGTGAACGGATTTG, 5′-3′TGTAGACCATGTAGTTGAGGTCA.

### Statistical methods

All data were presented as means ± standard deviation (SD), median (range) for continuous variables. The two-way Anova with Bonferroni post-hoc test was performed for multiple-group analysis. All statistical tests were two-sided and significance levels were defined as P < 0.05. All statistical analyses were performed with the SPSS software package (Version 17.0, SPSS, and Chicago, IL, USA) and GraphPad Prism (Version 5.01) software.

## Results

### PA inhibits the proliferation and promotes the apoptosis of H9C2 cells

**Figure 2:**
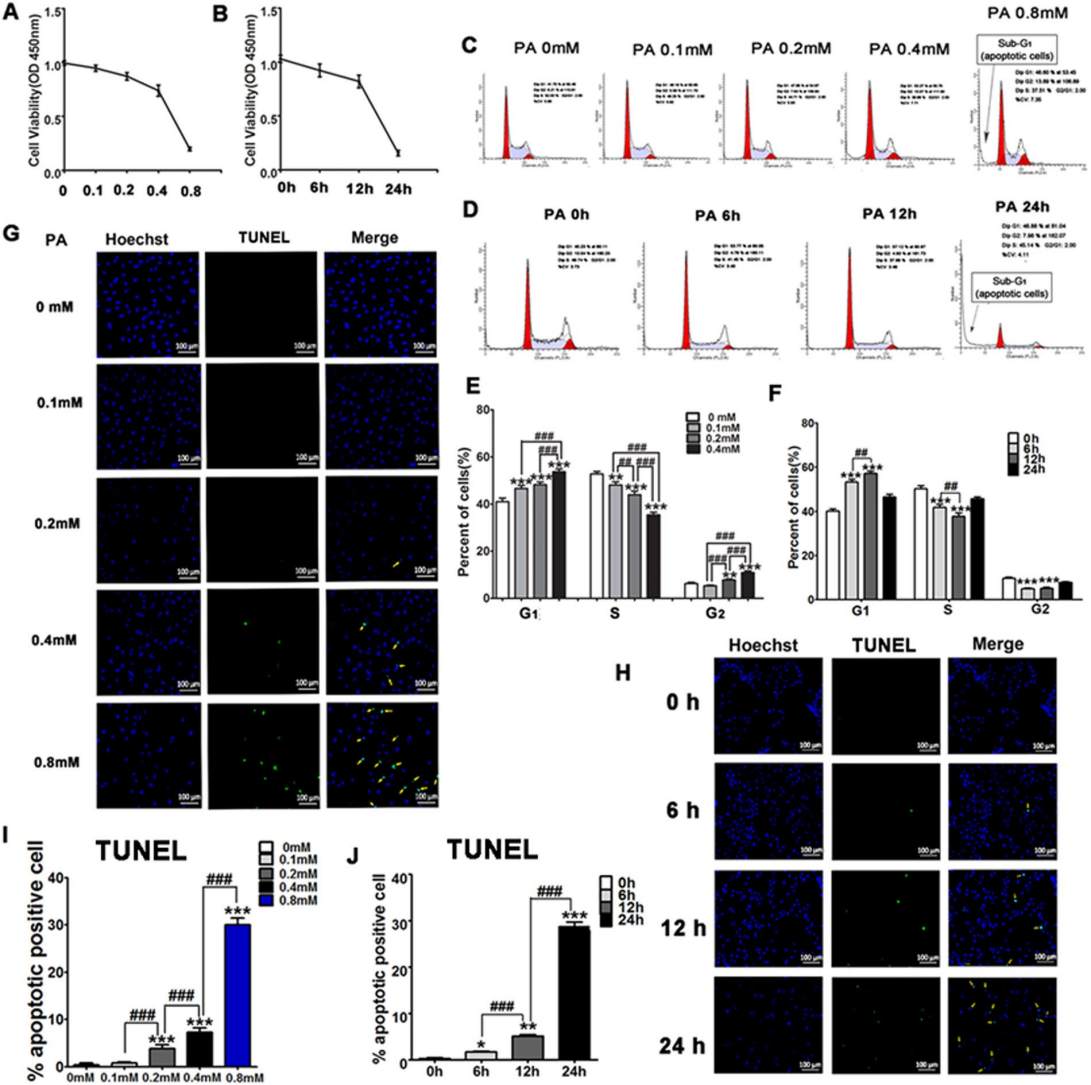
Palmitic acid inhibits H9C2 cell growth. The H9C2 cells were treated with different concentrations of palmitic acid (PA) for 12 hours or 0.4 mM PA at different time points. (**A,B**) Cell viability analyses of H9C2 cells using CCK-8 assay. (**C–F**) The cell cycle of H9C2 cells were detected by flow cytometry, and were analyzed by percent of H9C2 cells in different phases of distribution. (**G–J**) The apoptotic H9C2 cells were stained by TUNEL and then were examined under a fluorescence microscope (200×), scan bar = 100 μm. Cell apoptosis were analyzed by percent of apoptotic positive cells in each image. All data are representative of 3 independent experiments. The data are presented as mean ± SD (n = 3). *P < 0.05, **P < 0.01, ***P < 0.001 when compared with PA 0 mM or PA 0 hour.

H9C2 cells are derived from embryonic BD1X rat myocardium and exhibit many properties of primary cardiomyocytes including membrane morphology, protein expression of signaling pathways, electrophysiological properties, and hypertrophic response, though H9C2 cells are not able to beat. We used H9C2 cells to determine the role of PA, the major form of fatty acids, on cardiomyocytes viability. We treated H9C2 cells with various concentration of PA for 12 hrs or with 0.4 mM PA for various time points and then measured cell viability using CCK8 kit. We found that cell viability decreased in a PA dose-dependent (Fig. 2A) or in a time-dependent manner (Fig. 2B). Thus, PA suppressed cell viability. Further, we treated H9C2 cells with 0.4 mM PA for various time points or with various concentration of PA for 12 hrs as described above and analyzed cell cycle distribution by Flow Cytometry. Similar to the effect on cell viability, we observed that the number of H9C2 cells in G_1_ and G_2_ phases was increased and decreased in S phase in the PA-treated group in both dose-dependent (Fig. 2C,E) and time-dependent (Fig. 2D,F) manners, suggesting that PA suppresses H9C2 cells proliferation, leading to a decrease of cell viability. In addition to cell proliferation, we found an obvious sub-G_1_ phase resulting from apoptotic H9C2 cells treated with 0.8 mM PA for 12 hrs (Fig. 2C) or with 0.4 mM PA for 24 hrs (Fig. 2D), suggesting that PA may also promote cell apoptosis.

To confirm the induction of apoptosis by PA, we treated H9C2 cells with PA and then analyzed apoptotic cells with TUNEL. We found that PA treatment significantly increased TUNEL positive cells dose-dependently (Fig. 2G,I) and time-dependently (Fig. 2H,J), indicating that PA treatment promotes H9C2 cells apoptosis. Thus, PA treatment induced the reduction of H9C2 cell viability through both suppression of cell proliferation and promotion of cell apoptosis.

### PA influences autophagic flux in H9C2 cells by suppressing the conversion of autophagosomes to autophagolysosomes

**Figure 3:**
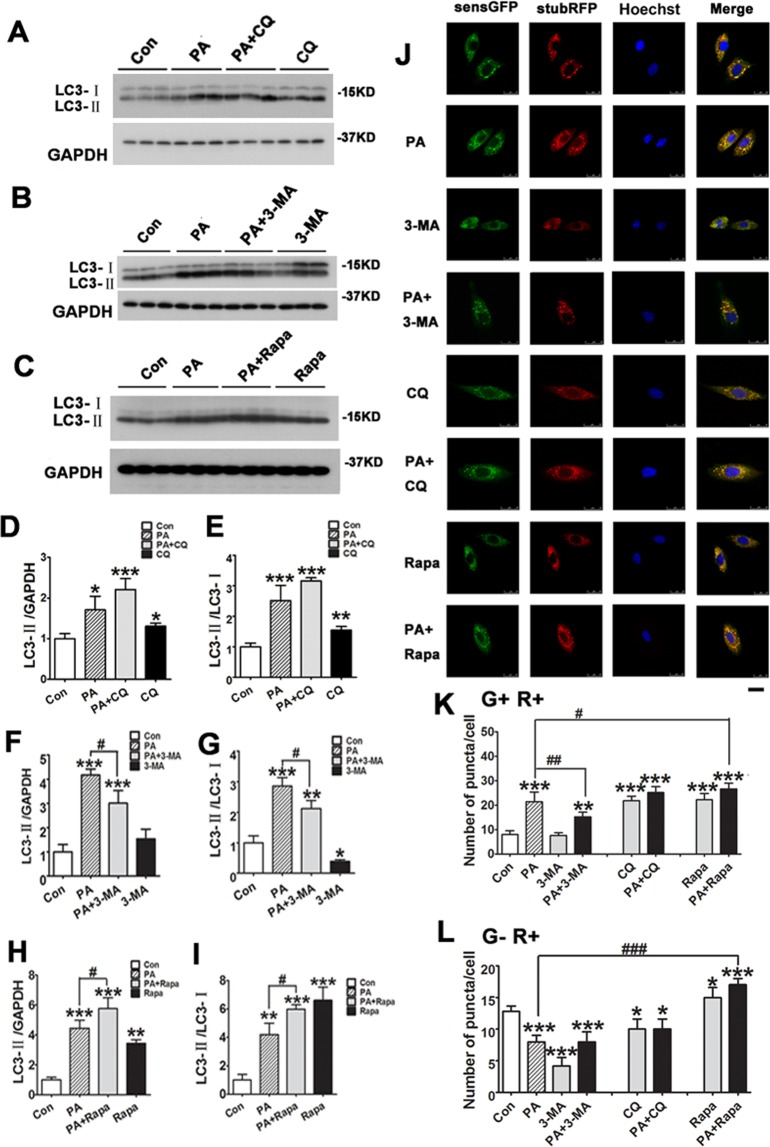
The effect of PA on autophagic flux in H9C2 cells. (**A–C**) H9C2 cells were treated with 0.4 mM PA for 12 hours in the absence or presence of chloroquine (CQ, 10 μM), 3-methyladenine (3-MA, 10 mM) or rapamycin (Rapa, 1 μM) for 2 hours, and then analyzed by Western blots. (**D–I**) Densitometric analysis of LC3-II/GAPDG and LC3-II/LC3-I expression by Western blots. (**J–L**) H9C2 cells stably expressing stubRFP-sensGFP-LC3 were pretreated with CQ, 3-MA or Rapa for 2 hours, followed by 0.4 mM PA for 12 hours, and then representative images from the confocal fluorescence (J, Scan bar = 25 μm) and the number of puncta (autophagosomes G + R+, and autophagolysosome G − R+) were quantified for each experiment (**K,L**). All data are representative of 3 independent experiments. The data are presented as mean ± SD (n = 3). *P < 0.05, **P < 0.01, ***P < 0.001 vs. Con group, ^#^p < 0.05, ^##^P < 0.01, ^###^p < 0.001.

Increasing evidence has demonstrated that altered cardiac autophagy is associated with cardiac injury in patients who have obesity^4,5^. Autophagic flux was measured by levels of the LC3-II, the conversion of LC3-I to LC3-II, the number of autophagosomes and autophagolysosomes, and levels of p62 which is inversely related to autophagic activity^9,10^. To study the role of PA in autophagic flux, we treated H9C2 cells with 0.4 mM PA for 12 hrs and then analyzed the levels of LC3-II by Western blots. We found that PA significantly induced LC3-II accumulation (Fig. 3A,D) and increased ratio of LC3-II/LC3-I (Fig. 3A,E), suggesting PA may affect autophagic flux by increasing autophagosome formation. To examine autophagosome turnover in H9C2 cells, we overexpressed stubRFP-sensGFP-LC3 in H9C2 cells virally and treated the cells with 0.4 mM PA for 12 hrs. We found that PA treatment increased the autophagosome accumulation (yellow puncta, G + R+) and decreased the autophagolysosome puncta (red puncta, G − R+) (Fig. 3J–L), suggesting PA may affect autophagic flux by increasing autophagosome formation but decreasing autophagolysosome, supporting that PA may initiate autophagosome formation or suppress the conversion of autophagosome to autophagolysosome.

Then, we influenced autophagy flux pharmacologically and determined the role of PA in autophagy in H9C2 cells. We pre-treated H9C2 cell with lysosomal acidification inhibitor CQ (10 μM) to block the degradation of existing autophagosomes (downstream step of autophagy) for 2 hrs and then added 0.4 mM PA into the cells for 12 hrs. Levels of LC3-II, LC3-I and LC3-II were analyzed by Western blots. We found that the increase in LC3-II accumulation in PA-treated H9C2 cells was not further enhanced by CQ addition analyzed by Western blot analysis (Fig. 3A,D,E). Moreover, the increased number of autophagosome puncta and autophagolysosome puncta were not increased further upon CQ pre-treatment compared with non-CQ pre-treated H9C2 cells (Fig. 3J–L). Thus, these data support PA treatment did not initiate the autophagosome formation in the early stage of autophagy.

Next, we assessed turnover of autophagy flux following incubation with PA for 12 hrs in the presence or absence of 10 mM 3-MA pre-treatment for 2 hrs which blocks autophagy activity. We found 3-MA significantly suppressed the PA-induced formation of autophagosomes (yellow puncta, G + R+) with no statistically significant difference in autophagolysosome (red puncta, G − R+) in H9C2 cells expressing stubRFP-sensGFP-LC3 (Fig. 3J–L). The effect was further confirmed by a Western blot analysis of LC3-II expression and the ratio of LC3-II/LC3-I in H9C2 cells (Fig. 3B,F,G). Thus, 3-MA which blocks neo-autophagosome and autophagolysosome, could not ameliorate the suppressed conversion of autophagosome to autophagolysosome.

Rapamycin (Rapa) activates autophagy. We then pre-treated H9C2 cells with 0.1 μM Rapa for 2 hrs followed with 0.4 mM PA treatment for 12 hrs. We found that Rapa significantly increased ratio of LC3-II expression, LC3-II/LC3-I expression, and induces the accumulation of autophagosomes (yellow puncta, G + R+) and autophagolysosome (red puncta, G − R+) in H9C2 cells compared with the control group (Fig. 3C,H–L). PA-induced accumulation of LC3-II and increase of LC3-II/LC3-I ratio were further increased in H9C2 cells by Rapa pretreatment. Thus, PA-induced accumulation of autophagosome (yellow puncta, G + R+) was further increased, and PA-induced decreased autophagolysosome puncta (red puncta, G − R+) was restored (Fig. 3C,H–L) by Rapa pre-treatment.

### PA suppresses the function of autophagy on p62 degradation

**Figure 4:**
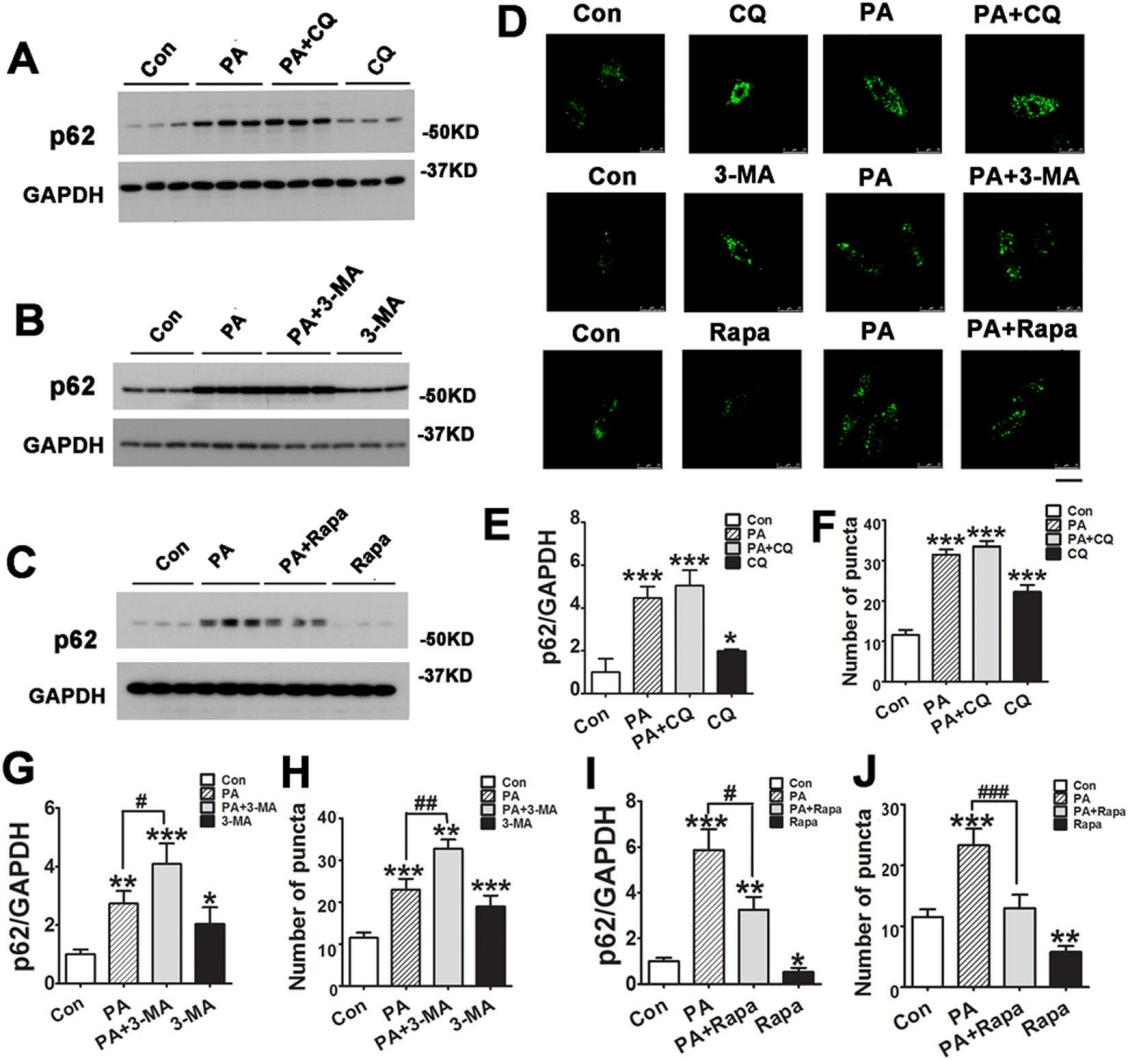
The effect of PA on autophagic degradation in H9C2 cells. (**A–C**) H9C2 cells were treated with 0.4 mM PA for 12 hours in the absence or presence of chloroquine (CQ, 10 μM), 3-methyladenine (3-MA, 10 mM) or rapamycin (Rapa, 1 μM) for 2 hours, then the cell lysates were then analyzed by Western blots. (**E,G** and **I**) Densitometric analysis of p62 expression by Western blots. (**D,F,H** and **J**) H9C2 cells stably expressing GFP-p62 were pretreated with CQ, 3-MA, or Rapa for 2 hours, followed by 0.4 mM PA for 12 hours. Then representative images from the confocal fluorescence (**D**, Scan bar = 25 μm) and quantitative analysis of the number of GFP-p62 puncta were calculated for each experiment. Each group of data is representative of 3 independent experiments. The data are presented as mean ± SD (n = 3). *P < 0.05, **P < 0.01, ***P < 0.001 vs. Con group, ^#^p < 0.05, ^##^p < 0.01, ^###^p < 0.001.

P62 is degraded by autophagic system. To determine the role of PA in autophagy function, we treated H9C2 cells with 0.4 mM PA for 12 hrs and analyzed p62 expression by Western blots and accumulation of GFP-p62 in cultured cells. We found that PA treatment led to an increase in p62 level and an accumulation of GFP-p62 positive puncta relative to the control group in H9C2 cells expressing GFP-p62 protein (Fig. 4A,D–F). These data suggest that PA may suppress autophagy degradation. PA induced increase of p62 expression and accumulation of GFP-p62 puncta were not enhanced further or reversed by CQ pre-treatment (Fig. 4A,D–F), supporting that PA induced autophagosome accumulation not by initiating the autophagy, but impairing autophagosome clearance by suppressing autophagolysosome degradation in H9C2 cells. Moreover, increases in p62 protein and the number of GFP-p62 puncta fluorescence in response to PA treatment were significantly augmented further by 3-MA pre-treatment in H9C2 cells (Fig. 4B,D,G,H), and significantly decreased by Rapa pre-treatment in H9C2 cell (Fig. 4C,D,I,J). Thus, these data suggest that 3-MA treatment did not improve the depressed autophagic degradation though it decreased autophagosome accumulation. Rapa pretreatment could attenuate lipid-impaired autophagic degradation and promote the autophagy flux smoothly.

### Selenate supplementation attenuates high-fat diet induced hyperlipidemia and hyperglycemia with slightly affecting body weight and blood glucose level

**Figure 5:**
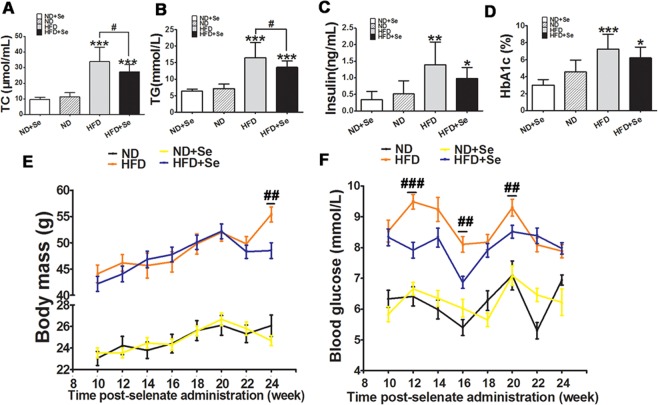
HFD-induced metabolic disorders was partially recovered by selenate administration in C57BL/6J mice. (**A–D**) Levels of total cholesterol (TC), triglyceride (TG), insulin, glycated hemoglobin A1c (HbA1c) in 4 groups of mice. (**E**) Level of body mass at different length time post-selenate administration in 4 groups in mice. (**F**) Level of blood glucose at different length time post-selenate administration in 4 groups of mice. The data are presented as mean ± SD (n = 15). *P < 0.05, **P < 0.01, ***P < 0.001 when versus ND group. ^#^p < 0.05, ^##^p < 0.01, ^###^p < 0.001 HFD + Se versus HFD group.

To investigate whether selenate suppressed obesity-associated metabolic disorders, we fed C57BL/6J mice with a normal rodent chow diet and a rodent HFD supplemented with or without sodium selenate for 24 weeks. All animals’ fasting body mass, total cholesterol (TC), triglyceride (TG), insulin, glycated hemoglobin A1c (HbA1c) were collected and measured by ELISA. We found that the body mass, blood glucose, TC, TG, insulin, and HbA1c were significantly higher in mice fed with HFD than those fed with ND (Fig. 5A–F). Selenate supplementation mitigated hyperlipidemia (Fig. 5A,B) and slightly decreased the levels of insulin and HbA1c induced by HFD (Fig. 5C,D), suggesting a protective role of selenate on HFD-induced dysregulation of lipid metabolism. Moreover, we found a significant decrease in body mass at post-selenate treated 24 weeks and in levels of blood glucose at post-selenate treated 10 to 20 weeks in HFD + Se group by comparison with untreated HFD group (Fig. 5E,F). These results confirm that selenate supplementation might attenuate HFD induced metabolic disruption.

### Selenate supplementation attenuates HFD-induced hypertrophic remodeling, fibrosis accumulation and myocardial dysfunction in C57BL/6J mice

**Figure 6:**
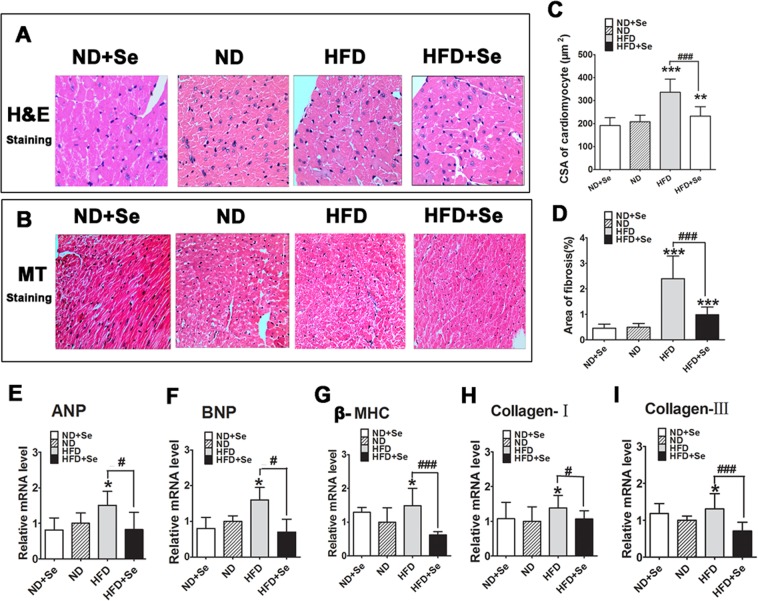
Selenate therapy mitigated HFD-induced cardiac hypertrophy and myocardial fibrosis in C57BL/6J mouse hearts. (**A**) Representative images of H&E staining with original magnification (×400) exhibiting cross-sectional area (CSA) of cardiomyocytes in each group (n = 5). (**B**) Representative images of Masson’s trichrome (MT) staining exhibiting myocardial fibrosis at magnification ×200 in left ventricles in four groups (n = 4). (**C**) Quantitative analysis of CSA with measurements of 30 cardiomyocytes in each section from 4 groups. (**D**) Quantitative analysis of percent area of myocardial interstitial fibrosis with normalizing blue MT staining area to total myocardial area from 10 randomly-selected fields in each section from four groups. (**E–I**) Changes in relative mRNA levels of ANP, BNP, β-MHC, collagen-I, and collagen-III in myocardium from four groups of mice (n = 6). The data are presented as mean ± SD. *P < 0.05, **P < 0.01, ***P < 0.001 when versus ND group. ^#^p < 0.05, ^##^p < 0.01, ^###^p < 0.001 HFD + Se vs. HFD group.

We randomly selected five mouse hearts in each group for evaluating cardiac histological preformation. Mice hearts were weighed and placed in 4% formaldehyde for histological study C57BL/6J mice. We found the CSA of cardiomyocytes was markedly increased in the HFD group compared to the ND group, indicating HFD-induced cardiomyocytes hypertrophy (Fig. 6A,C), this was done by quantitative analysis of H&E staining images. As determined by quantitative analysis of MT staining images, we found an elevated percent area of myocardial collagen in the HFD group compared with ND group (Fig. 6B,D), suggesting interstitial collagen accumulation caused by HFD exposure. Moreover, mRNA expression levels of ANP, BNP, β-MHC, Collagen I, and Collagen III were significantly higher in the HFD group as compared with the value in the ND group (Fig. 6E–I), suggesting increased mRNA expression caused by HFD exposure.

We found selenate treatment reversed cardiomyocytes’ morphology and significantly decreased the CSA of cardiomyocytes, compared with the HFD group (Fig. 6A,C). Selenate treatment reversed myocardial collagen deposition and notably decreased the percent area of fibrosis in the left ventricles, compared with the HFD group (Fig. 6B,D). Moreover, we found selenate addition by oral gavage remarkably suppressed HFD-induced relative increased mRNA levels of ANP, BNP, β-MHC, Collagen-I and Collagen-III. These findings suggest that HFD-induced cardiac hypertrophy and cardiac fibrosis accumulation could be attenuated by selenate administration.

### Selenate supplementation was protective against HFD-mediated cardiac autophagic degradation inhibition and reversed Akt phosphorylation induced by HFD in C57BL/6J mice

**Figure 7:**
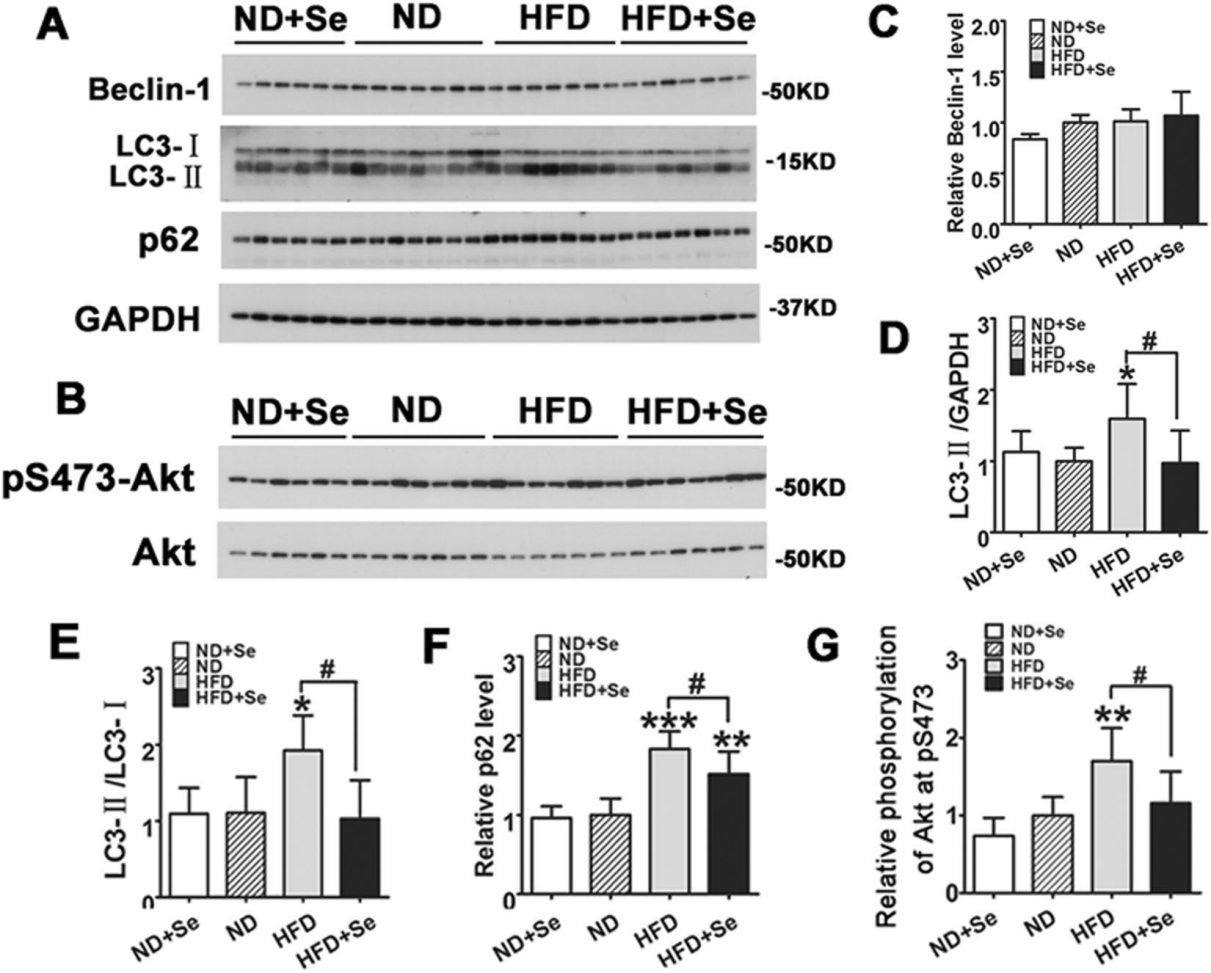
Selenate rescued cardiac autophagic degradation inhibition and Akt signaling when exposed to HFD in cardiac tissue of C57BL/6J mice. (**A,B**) Representative Western blot analysis of autophagic markers and Akt pathway in heart tissue of per group of C57BL/6J mice. (**C–G**) The column figures of Beclin-1, LC3- II, and p62 normalized by GAPDH; the ratios of LC3-II/LC3-I, phospho-Akt normalized by total Akt from the Western blots. The data are presented as mean ± SD (n = 6 - 7). *P < 0.05, **P < 0.01, ***P < 0.001 vs. ND group, ^#^p < 0.05, ^##^P < 0.01, ^###^p < 0.001 HFD^+^Se vs. HFD group.

We have demonstrated that PA-induced suppression in autophagic degradation and cell growth could be improved by Rapa due to improved autophagic degradation. In order to test cardiac autophagic changes in C57BL/6J mice, we measured protein markers of autophagic activity by Western blots, and found that HFD led to an increase in levels of LC3-II/LC3-I, and notably induced accumulation of LC3-II and cardiac p62 expression without significantly affecting expression of Beclin-1. This was observed through comparison with the ND group, and it suggests that cardiac autophagic degradation was broken off by HFD in mice (Fig. 7A,C–F). Then, we attempted to evaluate the effects of selenate supplementation on altered autophagic activity by analyzing the levels of LC3-II/LC3-I and p62 in C57BL/6J mice hearts of four groups. We found selenate supplementations significantly inhibited HFD-induced accumulation of LC3-II, level of LC3-II/LC3-I, and it markedly reversed p62 expression in response to HFD, demonstrating HFD-induced obstructed autophagic degradation and accumulated LC3-II were restored partially after selenate treatment. Instead, selenate addition did not affect Beclin-1 compared to that in the ND group (Fig. 7A,C–F).

It has been long been assumed that Akt and it’s cascades signaling are involved in the regulation of cell proliferation, survival, and metabolism. Akt signaling pathway mediates cardiac hypertrophy and interstitial fibrosis, as well as cardiac autophagy^11–17^. To further examine mechanisms of selenate in protecting against hyperlipidemia-induced cardiomyopathy, we performed the same experiments in C57BL/6J mice and noted a regulatory role of Akt signaling cascades by Western blots. We observed Akt activity was activated in HFD group compared to ND group, as markedly representive increased level of phospho-Akt at Ser^473^ by Western blots. As expected, the presence of selenate supplementation actively inhibited HFD-induced phosphorylation of Akt at Ser^473^ normalized with total Akt by Western blots (Fig. 7B,G). Taken together, selenate play a critical role in the process of regulation activity of Akt pathway after HFD exposure.

### Selenate ameliorates PA-mediated autophagy impairment and Akt activity in H9C2 cells

**Figure 8:**
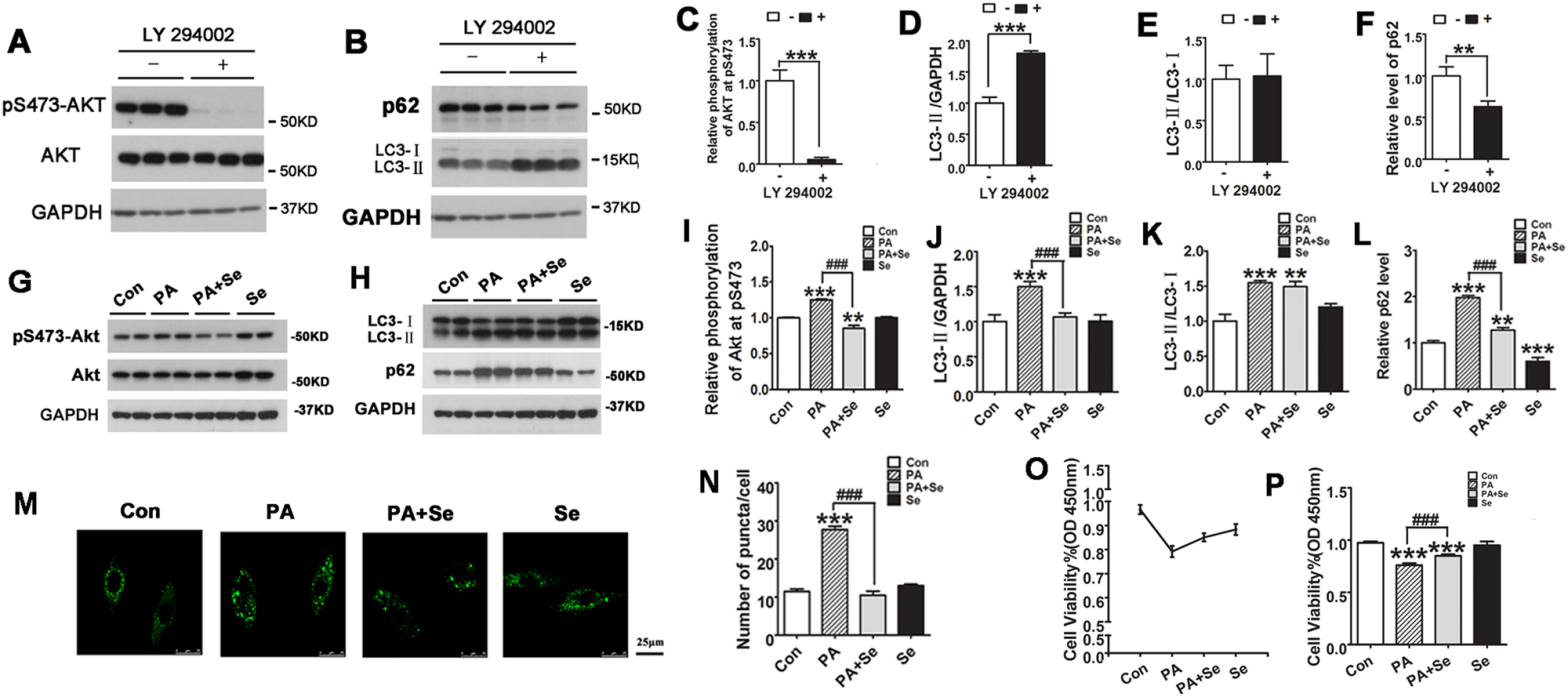
Selenate reversed cardiac autophagic degradation inhibition and regulated the activity of Akt when exposed to PA in H9C2 cells. (**A–F**) H9C2 cells were treated with 10 μM LY294002 for 12 hours. Representative Western blots images and of densitometric analysis of the relative levels of pS473-Akt, LC3-II, p62 and the ratio LC3-II to LC3-I expression in H9C2 cells. (**G–L**) H9C2 cells were treated with 0.4 mM PA for 12 hours in the absence or presence of 0.5 μM of the selenate (Se) for 12 hours. Representative Western blots images and densitometric analysis of phospho-Akt, Akt, LC3-II, LC3-II/LC3-I and p62 expression in H9C2 cells. (**M,N**) H9C2 cells stably expressing GFP-p62 were pretreated with Se for 12 hours, followed by 0.4 mM PA for 12 hours, and then representative images from the confocal fluorescence (M, Scan bar = 25 μm) and quantitative analysis of number of GFP-p62 puncta. (**O,P**) H9C2 cells were treated with 0.4 mM PA for 12 hours in the absence or presence of 0.5 μM of Se for 12 hours. Cell viability analyses of H9C2 cells using CCK-8 assay. The experiments were repeated for 3 times. All data are presented as mean ± SD (n = 3). *P < 0.05, **P < 0.01, ***P < 0.001 vs. Con group, ^#^p < 0.05, ^##^P < 0.01, ^###^p < 0.001.

To determine the molecular signaling pathway that regulates autophagy in H9C2 cells, we analyzed Akt activity by Western blots, which plays an important role in autophagy. We treated the H9C2 cells with LY294002, the inhibitor of PI3K, which further decrease the phosphorylation and activity of Akt. We found a remarkable LC3-II accumulation and a decrease of p62 expression when phosphorylation levels of Akt at Ser473 was decreased measured by Western blots in the LY294002 treated H9C2 cells compared to the control treated cells (Fig. 8A–F). The data suggest that the inhibition of Akt phosphorylation after LY294002 exposure initiates autophagic degradation.

To further elucidate whether selenate protections against hyperlipidemia-induced cardiac injury, we treated H9C2 cells with 0.5 μM selenate before exposure to PA. We found that selenate pretreatment significantly decreased PA- induced LC3-II accumulation in H9C2 cells (Fig. 8H,J,K). Moreover, selenate pretreatment could attenuate PA-induced accumulation of p62 protein by densitometric analysis of Western blot and decrease number of GFP-p62 puncta by quantitative analysis of number of GFP-p62 puncta (Fig. 8H,L–N). These data suggest that selenate ameliorates PA-induced autophagy impairment and maintain autophagy influx in status of cardiomyocytes. To learn the molecular mechanism involved in the attenuation of PA-induced autophagy impairment by selenate, we analyzed Akt activity by Western blots in above PA and selenate-treated H9C2 cells. We found that phosphorylation levels of Akt at Ser473 were increased in the cells treated with 0.4 mM PA compared to the control treated cells, suggesting the activation of Akt signaling in H9C2 cells by PA. Selenate pretreatment attenuated PA-induced increase of phospho-Akt (Fig. 8G,I). Furthermore, selenate pretreatment improved significantly reduction of H9C2 cell viability induced by PA (Fig. 8O,P). Taken together, these findings suggest that selenate ameliorates PA induced autophagy impairment and reduction of cell viability in H9C2 cells, which is associated with regulation of the process of autophagic degradation and regulation activity of Akt pathway after lipid exposure.

## Discussion

Obesity has become one of the most prevalent metabolic diseases all over the world. In patients with obesity, the crucial causes of morbidity and mortality are cardiovascular disorders. So far, the effective treatments for obesity-induced cardiac structure remodeling and dysfunction are not identified. In this study, we demonstrated that long-term HFD would lead to hyperlipidemia, enhance blood glucose/HbA1c, and induce hypertrophic cardiomyocytes and cardiac fibrosis deposition in C57BL/6J mice. We also found diet containing selenate dramatically ameliorated lipid metabolism, reversed HFD-induced hypertrophic cardiomyocytes and cardiac fibrosis accumulation. In addition to these findings, it was observed that selenate supplementation ameliorated HFD-induced cardiac autophagic degradation inhibition and reversed HFD-activated phosphorylation of Akt. It is possible to conclude that selenate administration contributes mainly with a cardio-protective role in obesity-related cardiac abnormalities by regulating cardiac autophagic degradation and Akt pathways. These results confirm that selenate plays an important role in ameliorating hyperlipidemia, myocardial hypertrophy, cardiac fibrosis deposition, and ultimately prevention of heart remodeling in mice.

The process of autophagy is mainly activated in response to external stressor, and is characterized by the formation of autophagosomes; subsequently, autophagosomes target and fuse with lysosome to degrade engulfed damaged proteins or organelles in order to maintain intracellular homeostasis, differentiation, and survival^24^. Previous studies have showed that changed autophagy is correlated with regulating HFD/PA-associated cardiac hypertrophy, cardiac and intracellular Ca^2+^ derangements^4,5,25^, especially autophagy is inhibited in metabolic cardiomyopathy^26^. Moreover, the activation of autophagy in the heart has contributed to ameliorate hypertrophic cardiomyopathy, alleviate cardiac dysfunction and myocardial injury^27–30^. Furthermore, there is evidence indicated that selenium compounds can exert both stimulating and inhibitory effects on autophagy, as well as play both ameliorative or adverse effects on oxidative stress and the maintenance of health, depending on the environmental stress, diseases, cell lines, selected selenium compounds, and the presence of applied concentration^31–34^. Accordingly, we postulate cardio-protective effects of selenate are closely connected with autophagy changes. In our study, we found inhibited autophagic degradation under high-lipid conditions, and treatment of selenate in an applied concentration successfully rescued HFD-induced suppressed autophagic degradation in mice. Thus, selenate limited HFD-induced cardiac remodeling and cardiac injury probably through modulating relatively levels of autophagy.

The exact mechanism that regulates autophagy and cardiac remodeling remains to be explained. It has been shown that the Akt is essential for controlling cardiac autophagy and autophagy-related apoptosis in the heart tissue^3,4,29,30^. Moreover, activated Akt promotes cardiac hypertrophy under pressure overload or β-adrenergic receptor stimuli in transgenic mice^13^. Alternatively, in cardiomyocytes, prolonged Akt activation results in pathological hypertrophy with an increase in interstitial fibrosis^17^. It also has been indicated that selenium compound could inhibit Akt phosphorylation and its down effectors which is involved in modulating autophagy^34^. In our study, we found increased phosphorylation of Akt after lipid overload, whereas it was dramatically decreased after selenate supplement, accompanied by altered CSA of cardiomyocytes and cardiac fibrosis in C57BL/6J mice, as well as H9C2 cells viability. In addition, we provided important information that regulation of phosphorylation in Akt pathway accompanied by fluctuations of p62 expression. These results suggest that the Akt pathway plays an essential role in mediating HFD-associated cardiac structure changes and disrupted autophagic degradation, which provide further evidence that the ameliorative effects of selenate on cardiac remodeling are through regulating Akt pathway and autophagic degradation, providing a molecular mechanisms of explanation for autophagy, and a potential causative relationship between the two.

Nonetheless, a few unresolved issues remain. First, although we have established HFD-induced cardiac injury by histologic data, we did not present data of echocardiological measurements. Then, we did not measure GP_X_1 activity or expression in myocardium. Last, it is not explained in detail why inhibition of cardiac autophagy appears to be the reason of HFD-induced cardiac injury. It will be of interest to provide more data that autophagy inhibition contributes to HFD-induced myocardial injury or autophagy restoration reduces the cardiac impairments.

In summary, our data turn out that selenate treatment ameliorates hyperlipidemia, and attenuates cardiomyocytes hypertrophy and deposition of fibrosis, together with improved cardiac function. The selenate’s benefits are closely related to the regulating activity of Akt signaling pathway and cardiac autophagic degradation. Treatment with selenate or in combination with other therapy might be an attractive option against obesity-induced metabolic disorders and cardiac injury.

## Data Availability

All data generated or analyzed in the current study are included in this published article.
